# Evaluation of FRET real-time PCR assay for rapid detection and differentiation of *Plasmodium *species in returning travellers and migrants

**DOI:** 10.1186/1475-2875-7-70

**Published:** 2008-04-28

**Authors:** Innocent Safeukui, Pascal Millet, Sébastien Boucher, Laurence Melinard, Frédéric Fregeville, Marie-Catherine Receveur, Thierry Pistone, Pierre Fialon, Philippe Vincendeau, Hervé Fleury, Denis Malvy

**Affiliations:** 1 Pôle de Biologie Moléculaire, CHU de Bordeaux, Hôpital Pellegrin, Place Amélie Raba Léon, 33076 Bordeaux Cedex, France; 2 Laboratoire de Parasitologie, CHU de Bordeaux, 1 rue Jean Burguet, 33071 Bordeaux Cedex France; 3 Centre René Labusquière, Université Victor Segalen Bordeaux 2,146 rue Léo Saignat, 33076 Bordeaux Cedex, France; 4 Travel clinic and Imported Diseases Units, Department of Internal Medicine, University Hospital Centre, 1 rueJean-Burguet, 33071 Bordeaux Cedex, France; 5 Safeukui Noubissi Innocent, Institut Pasteur, Unité d'Immunologie Moléculaire des Parasites, URA CNRS 2581, 28 Rue du Dr Roux, 75075 Paris, France

## Abstract

**Background:**

A simple real-time PCR assay using one set of primer and probe for rapid, sensitive and quantitative detection of *Plasmodium *species, with simultaneous differentiation of *Plasmodium falciparum *from the three other *Plasmodium *species (*Plasmodium vivax, Plasmodium ovale *and *Plasmodium malariae*) in febrile returning travellers and migrants was developed and evaluated.

**Methods:**

Consensus primers were used to amplify a species-specific region of the multicopy 18S rRNA gene, and fluorescence resonance energy transfer hybridization probes were used for detection in a LightCycler platform (Roche). The anchor probe sequence was designed to be perfect matches to the 18S rRNA gene of the fourth *Plasmodium *species, while the acceptor probe sequence was designed for *P. falciparum *over a region containing one mismatched, which allowed differentiation of the three other *Plasmodium *species. The performance characteristics of the real-time PCR assay were compared with those of conventional PCR and microscopy-based diagnosis from 119 individuals with a suspected clinical diagnostic of imported malaria.

**Results:**

Blood samples with parasite densities less than 0.01% were all detected, and analytical sensitivity was 0.5 parasite per PCR reaction. The melt curve means Tms (standard deviation) in clinical isolates were 60.5°C (0.6°C) for *P. falciparum *infection and 64.6°C (1.8°C) for non-*P. falciparum *species. These Tms values of the *P. falciparum *or non-*P. falciparum *species did not vary with the geographic origin of the parasite. The real-time PCR results correlated with conventional PCR using both genus-specific (Kappa coefficient: 0.95, 95% confidence interval: 0.9 – 1) or *P. falciparum*-specific (0.91, 0.8 – 1) primers, or with the microscopy results (0.70, 0.6 – 0.8). The real-time assay was 100% sensitive and specific for differentiation of *P. falciparum *to non-*P. falciparum *species, compared with conventional PCR or microscopy. The real-time PCR assay can also detect individuals with mixed infections (*P. falciparum *and non-*P. falciparum *sp.) in the same sample.

**Conclusion:**

This real-time PCR assay with melting curve analysis is rapid, and specific for the detection and differentiation of *P. falciparum *to other *Plasmodium *species. The suitability for routine use of this assay in clinical diagnostic laboratories is discussed.

## Background

*Plasmodium falciparum *and *Plasmodium vivax *account for the majority of malaria cases worldwide [1]. *Plasmodium falciparum *is responsible for complications and death, mainly in non-immune individuals. On the other hand, *P. vivax *and *Plasmodium ovale *induce mild malaria symptoms restricted to fever with rare complications, while *Plasmodium malariae *infections result in mild symptomatic malaria. Furthermore, *P. vivax *and *P. ovale *produce dormant liver stages that may result in relapse of infection months to years later, while *P. malariae *infections can persist for decades, although dormant liver forms are not thought to occur.

It is currently estimated that 50–80 million individuals from industrialized countries visit malaria-endemic areas each year and approximately 10,000–30,000 travellers contract malaria [2], among which severe malaria accounts for approximately 5% (range 1–38%) [3], with a mortality rate ranging from 0.6% to 3.8% mainly resulting mostly from late and/or misdiagnosis, and delayed treatment administration [4-8]. Because about 90% of travellers who contract malaria will not become ill until returning home, preventing malaria-associated morbidity and mortality requires improved rapid and accurate laboratory diagnostic tools detecting low parasitaemia and differentiating febrile patients with *P. falciparum *from the other *Plasmodium *species. Such diagnostic, performed at the time of patient admission, will allow a prompt and adequate treatment and follow-up [9].

Light microscopy of thick and thin Giemsa-stained blood smears remains the gold standard for malaria diagnostic [10]. However, even in expert hands (increasingly missed in industrialized countries), microscopy demonstrated limitations, mostly related to low sensitivity (detection limit: 10–50 trophozoites/μl) and misdiagnosis [4,11-15]. In some case, parasite morphology is damaged due to exposition to prophylactic medication or auto-medication, making malaria biological diagnosis more difficult.

Alternative methods for laboratory diagnostic of malaria have been developed, including fluorescence microscopy of parasite nuclei stained with acridin orange and rapid dipstick immunoassays. The advantages offered by these methods, such as the fact that a result can be obtained within half an hour by non-skilled technicians, are tempered by three limitations reviewed by Moody et *al *[16]: 1) the dipstick tests do not improve sensitivity over microscopy and the sensitivity decreases as parasitaemia falls below 100 parasites/μl [17]; 2) false positives are observed, particularly after treatment, as the parasite antigens detected can remain in the circulation following parasite clearance, or in the presence of pneumococcal meningitis infection [18]; and 3) many dipstick tests are specific to *P. falciparum *infections.

A variety of PCR-based techniques have been developed for the genus or species-specific diagnosis of malaria parasite infection [11,13,19-21]. While demonstrating increased sensitivity and specificity, they remained labour-intensive, time-consuming and prone to carry more DNA contamination during manipulation of post-amplification products. The recent advance of a real-time quantitative PCR technique has proven usefulness in various applications, including parasite detection, species differentiation, gene expression and regulation, and allelic discrimination [22-30]. However, the large majority of developed real-time PCR assays used many set of primers and/or probes to analyse each sample, thus increasing cost and reliability.

To date, one fluorescence resonance energy transfer (FRET) real-time PCR assay using one set of primer and probe [31], and two others real-time PCR methods using one set of primer and SYBR green dye [26,32] for *Plasmodium *sp. identification and species differentiation have been evaluated. The principal limitation of these assays was the lack of sensitivity of parasite detection: two to 30 parasites/μl of blood according to the study. Furthermore, the real-time PCR approach using SYBR green dye did not avoid the quantification of non-specific amplification products [33]. Here, a real-time PCR assay using a single set of primer and FRET hybridization probe for sensitive and quantitative detection of *Plasmodium *species, with simultaneous differentiation of *P. falciparum *from other human *Plasmodium *species was developed and evaluated. Results from the real-time PCR assay were compared to conventional PCR methods and microscopy examination of blood smears.

## Methods

### Origin of the clinical specimens and DNA template preparation

A total of 119 blood samples were collected in EDTA tube from travellers or migrants admitted to the Saint André University Hospital in Bordeaux, with suspected clinical malaria from years 2000 to 2006. Blood samples were collected before the initiation of the antimalarial treatment. One aliquot of each sample was used for routine biological diagnostic in the Biology Laboratory and the other one was stored at -80°C until use. DNA was extracted from 200 μl of blood using either the QIAamp DNA mini Kit (QIAGEN) or using an automated DNA extraction machine (MAGNA PURE LC, Roche Diagnostics, Indianapolis, Ind.) according to the manufacturer's instructions. The samples were run blindly for real-time PCR and conventional PCR assays.

### Primer and probe design

**Figure 1:**
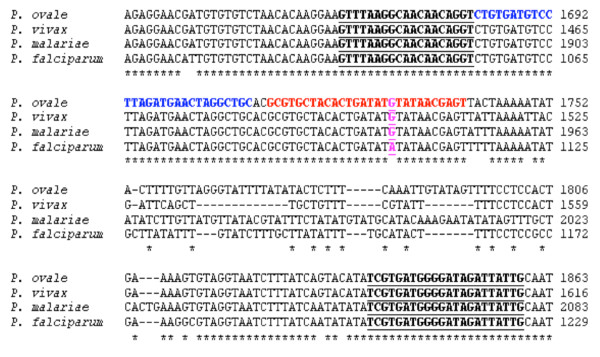
**Primer and fluorescence probe positions selected for FRET PCR of *Plasmodium *18S rRNA gene.** Sequences of forward (underline left panel) and reverse (underline right panel) primers were aligned with the corresponding target sequences. FRET probes for detection of parasite (blue and red for anchor and acceptor FRET hybridization probes, respectively). Acceptor FRET hybridization probe was designed on the basis of one nucleotide mismatch difference (shown in pink colour) that distinguish 18S rRNA gene of *P. falciparum *(GenBank Accession number: AL010278) from that of the *P. vivax *(GenBank Accession number: U83877) or *P. ovale *(GenBank Accession number: L48986) or *P. malariae *(GenBank Accession number: M54897).

The primers and probes were designed using the multicopy, stable and highly conserved [34] 18S rRNA gene single-stranded sequences of *Plasmodium *species available from GenBank (*P. falciparum*, accession number: AL010278; *P. vivax*, accession number: U83877; *P. ovale*, accession number: L48986 and *P. malariae*, accession number: M54897). The primers and probes are summarized in Table 1. Figure 1 shows their position and the alignment results of the 18S rRNA gene single-stranded sequences using the ClustalW multiple sequence alignment program (EMBL – European Bioinformatics Institute, Cambridge, UK). The sequences of the primers and the anchor FRET hybridization probe were designed to be perfect matches (i.e., 100% homologous) to the *P. falciparum*, *P. vivax*, *P. ovale *and *P. malariae *sequences. The sequence of the acceptor FRET hybridization probe was designed on the basis of one nucleotide mismatch difference that distinguish the 18S rRNA gene of the *P. falciparum *from those of the three other *Plasmodium *species. To ensure proper hybridization of the probe to the target sequence, oligonucleotides with a Tm at least 5°C higher than the actual annealing/extension temperature were chosen as probes. The 3' end of the anchor FRET hybridization probe was labelled with fluorescein isothiocyanate (FITC) and the 5' end of the acceptor FRET hybridization probe with LCRed705. Possible oligonucleotide dimers formation and/or self-complementarity and the theoretical melting temperatures of primers and probes (Tms) were calculated using Oligo programme version 4 (LightCycler Probe Design Software, Roche). Oligonucleotide primers and probes were obtained from Eurogentec and Proligo, respectively. The *P. falciparum*-specific primers were designed to amplify a 120-bp region from the cytochrome *c *oxidase subunit 1 (*cox1*) mitochondrial gene.

**Table 1:** Primers and probes selected for PCR of *Plasmodium *18S rRNA and cox1 genes

			Sequence	Target gene
Primers	Genus specific	Forward	5'-GTTTAAGGCAACAACAGGT-3'	18S rRNA
		Reverse	5'-CAATAATCTATCCCCATCACGA-3'	
	*P. falciparum specific*	Forward	5-TTACATCAGGAATGTTATTGC-3	cox1
		Reverse	5-ATATTGGATCTCCTGCAAAT-3	
Probes		Sensor	5'-ACTCGTTATACATATCAGTGTAGCACGC (FLU)-3'	18S rRNA
			5'-(Red 705)	
		Anchor	GCAGCCTAGTTCATCTAAGGACATCACAG (P)- 3'	

Amplicon length: *P. ovale*: 198 pb (1662 – 1859); *P. vivax*: 178 pb (1435 – 1612); *P. malariae*: 207 pb (1873 – 2079) and *P. falciparum*: 190 pb (1035 – 1225). The nucleotide positions are those reported in GenBank

### FRET real-time PCR

#### Amplification and detection

Purified DNA templates were amplified in a LightCycler analytical PCR system (version 6.0, Roche Diagnostics, Indianapolis, Ind.). The real-time PCR was performed using the "LightCycler DNA Amplification Kit Hybridization Probes" (Roche Diagnostics, Mannheim, Germany), according to the manufacturer's instructions. The reaction was realized in a final volume of 20 μl in each capillary tube containing 3.0 mM MgCl_2_, 0.5 μM of each primer, 0.25 μM of acceptor probe, 0.20 μM of anchor probe, 2 μl of 10× LightCycler-FastStart DNA master hybridization probe mixture, and 10 μl of extracted DNA. After a short centrifugation (700 *g *for 10 s), the sealed capillaries were placed into the LightCycler. The PCR program ran as follows: (i) 8 min at 95°C for enzyme activation and DNA denaturation, and (ii) 45 PCR amplification cycles consisting of 95°C for 15 s, 57°C for 10 s, and 72°C for 8 s. The temperature transition rate was 20°C/s in the denaturation and annealing steps and 5°C/s in the polymerization step. In the final cycle, the melting curve was obtained by initially heating to 95°C for 1.50 mn and subsequently cooling the samples to 45°C for 30 s. A final heating step to 95°C was realized with a controlled temperature transition rate of 0.4°C/s. The resulting fluorescence was recorded starting at the final 95°C heating step using the parameter "continuous mode" and the F3/F1 channel.

#### Interpretative criteria

The presence of an amplification or quantification curve for the LC705 signal captured in the F3 channel of the LightCycler, in conjunction with a melt curve with a melting temperature (Tm) of about 60.0°C, was considered a positive result for the *P. falciparum*; the presence of a quantification curve with a corresponding melt curve with a Tm of about 65.0°C was considered a negative result for the *P. falciparum *species and positive for at least one of the other three species. The absence of a quantification curve was considered as negative for the fourth species.

#### Analytical sensitivity

The minimum detection limit of the *Plasmodium *FRET assay was evaluated by use of a 10-fold dilution series of DNA (5000 to 0.25 parasites in a 20 μl PCR reaction volume) extract from blood samples recovered from four patients infected with *P. falciparum *(parasitaemia: 0.01% in Giemsa-stained thin blood smear). Each experiment included one reaction mixture without DNA as a negative control, and each specimen was run in duplicate for real-time PCR assay in parallel to conventional PCR.

#### Analytical specificity

To estimate the analytical specificity of the *Plasmodium *FRET assay, DNA was obtained and managed from European individuals who have never travelled to malaria endemic area (n = 10) or patients diagnosed with other infectious microorganisms (*Toxoplasma gondii*, *Leishmania infantum*, *Pneumocistis carinii*, *Loa loa *and *Trypanosoma brucei gambiense*, n = 2 samples for each microorganism).

#### Clinical specimen's sensitivity and specificity

The clinical sensitivity and specificity of the *Plasmodium *FRET assay for detecting and identifying malaria parasite species were calculated on 119 whole-blood samples, using conventional Giemsa-stained blood smear as the reference gold standard.

### Conventional PCR amplification

Two conventional PCR procedures were conducted: one with *Plasmodium *genus-specific primers (the same primers used in real-time PCR assay) and another with *P. falciparum*-specific primers. The PCR reaction was realized in a final volume of 50 μl containing the following reagent mixture: 1× of PCR buffer 10× (500 mM KCl, 100 mM Tris-HCl [pH 8.3], 20 mM MgCl_2_), 5 mM concentration of MgCl_2_, 0.5 μM each oligonucleotide primer, 200 μM deoxynucleoside triphosphate (Eurobio), and 0.02 unit *Taq *DNA polymerase (Eurobio). For each PCR tube, 40 μl PCR reagent mixture and 10 μl DNA were used. The reactions were carried out under the following conditions: (i) initial denaturation at 94°C for 3 mn, (ii) 35 cycles of: denaturation at 94°C for 30 s, annealing at 58.5°C for 30 s, extension at 72°C for 8 s, and (iii) final extension at 72°C for 5 min. Each experiment included one positive control (consisting of *P. falciparum *genomic DNA from positive blood samples) and one negative control (consisting of sterile double-distilled water). Amplified PCR products were detected by running 14 μl of the PCR mixture on a standard 1.5% agarose gel stained with a 1.0 μg/ml ethidium bromide solution and visualized under UV light.

### Statistical analysis

The 95% confidence intervals of proportions were calculated using the exact binomial test. Concordance between the results of real-time PCR and either conventional PCR assay or microscopy was analysed using the Kappa test [35]. All statistical analyses were performed using the SAS software (version 8.2).

## Results

### Detection of *Plasmodium* genus and species differentiation

**Figure 2:**
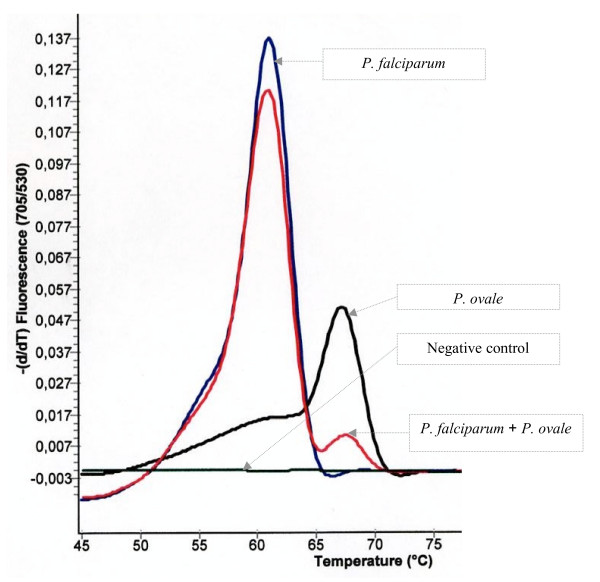
**Melting curves of amplicons post real-time PCR of DNA extracted from three different blood samples with known *P. falciparum*, non-*P. falciparum *(*P. ovale*) and mixed *Plasmodium *sp. (*P. falciparum *+ *P. ovale*).** The Tms of the *P. falciparum *were distinctively lower than that of *P. ovale*. Negative control included reaction mixture with water.

DNA extracted from blood samples recovered from seven febrile patients harbouring *Plasmodium *species (Giemsa-stained blood smear positive) was used as a template for this assay: patients presenting with single species infections diagnosed by microscopical examination (two patients with red blood cells harbouring *P. falciparum *or *P. vivax*, and one with *P. ovale *or *P. malariae*) and one patient with red blood cells harbouring both *P. falciparum *and *P. ovale*. Typical amplification plots (change in fluorescent signal versus cycle numbers) with a Ct varying from 24.8 to 31.8 were obtained. Conventional PCR with genus-specific primers (the same primers used for real-time PCR) and DNA sequence analysis confirmed the specific amplification of the 18S rRNA gene of *Plasmodium *species fragment. The FRET real-time PCR assay data allowed differentiating *P. falciparum *from the other three *Plasmodium *species. Mixed parasitism (*P. falciparum *plus non-*P. falciparum*) were identified by the presence of the two peaks simultaneously (Figure 2). The melting curve means Tms (standard deviation) were 61.0 (0.8)°C for *P. falciparum *(means of three patients, two harbouring *P. falciparum *only and the other with two *Plasmodium *species) and 67.0 (0.7)°C for the three other species (means of five individuals infected, four harbouring non-*P. falciparum *only and the other one mixed infection).

### Analytical sensitivity and specificity

**Figure 3:**
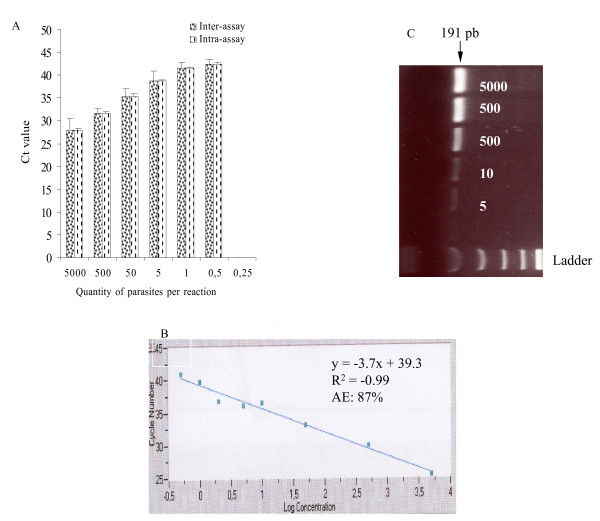
**Sensitivity, inter- and intra-assay variabilities of *P. falciparum *DNA quantification.** (A) Results obtained from four independent tests in duplicate by real-time PCR assay using FRET. (B) Linearity of FRET assay PCR is shown using serially diluted *P. falciparum *DNA (5000 to 0.25 parasites per reaction, one representative experiment). Amplification efficiency (AE) is calculated based on the slope of the standard curves using the formula: E = 10^1/-*s*^-1, where E(100) is the % efficiency and s is the slope of the standard cure. (C) Amplification of serially diluted *P. falciparum *DNA (5000 to 5 parasites per PCR reaction) by conventional PCR assay followed by gel electrophoresis and ethidium bromide staining.

The sensitivity, inter- and intra-assay variabilities for four independent experiments are shown in Figure 3A. Positive signals (Ct values) were found for all dilutions except for 0.25 parasite per reaction. A detection limit of 0.5 *P. falciparum *per PCR volume was achievable. The Ct mean values (standard deviations), ranging from 27.9 (2.0) for 5000 parasites per PCR volume to 42.6 (0.9) for 0.5 parasite per PCR volume. Amplification efficiency of real-time PCR reaction varied between 87 to 98% according to experiment (Figure 3B). A significant coefficient of correlation was found for the mean Ct values and *P. falciparum *concentration (r = -0.98) (Figure 3B). These DNA samples diluted were also amplified by conventional PCR with genus-specific primers and followed by electrophoresis and ethidium bromide staining (Figure 3C). The limit of detection was five parasites per PCR volume (Figure 3C). The conventional PCR assay was ten times less sensitive than the FRET real-time PCR assay. No PCR amplification by either real-time PCR, or conventional PCR with genus-specific primers was observed with any DNA samples obtained from European individuals who have never travel to malaria endemic area or patients infected with *T. gondii*, *L. infantum*, *P. carinii*, *L. loa *filaria or *T. brucei gambiense*.

### Clinical specimen sensitivity and specificity

One hundred and nineteen febrile patients with a suspected clinical diagnosis of imported malaria were included in this study. Asexual parasitaemia means [95% confidence interval or CI_95%_] at the inclusion was 0.7% [0.3–1.2] (Giemsa-stained blood smears microscopic examination). Two patients were gametocytaemic at day 0, among which one harboured only gametocytes without asexual stages. Thirty-eight, 34 and three patients returning from Central (Gabon, Republic of Central Africa, Congo and Cameroon), Western (Benin, Burkina Faso, Ivory Cost, Guinea, Senegal and Togo) and Eastern (Kenya and Mozambique) African countries, respectively. Other patients travelled in Madagascar (n = 4), French Guyana (n = 2), Comores (n = 1) and Thailand (n = 1). The geographical origin of parasites was not recorded for 36 patients.

**Figure 4:**
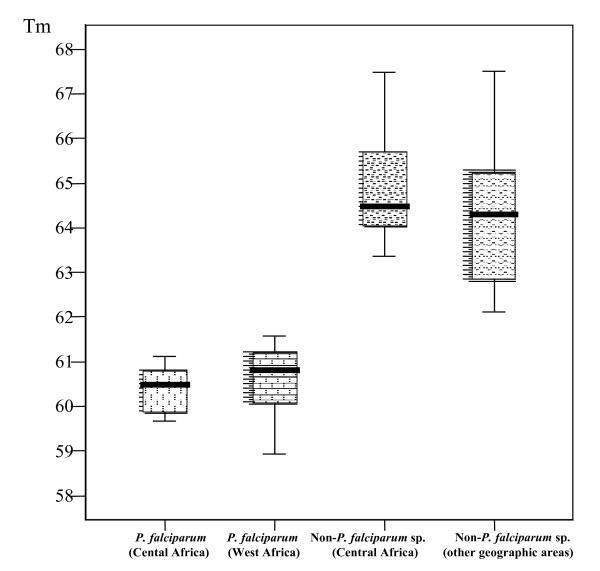
**Amplicon melting temperature plots (Tms) of *P. falciparum *and non-*P. falciparum *sp. according to the geographic origin of infection.** Tms of the *P. falciparum *or Non-*P. falciparum *species were similar between origin of parasite. n, effective.

Melting curve temperatures (Tms) of *P. falciparum *were defined as Tms of DNA samples that were positive by 18S real-time PCR with only one melting curve and positive by conventional PCR using both *genus*- and *P. falciparum*-specific primers. The Tms of non-*P. falciparum *species were defined as Tms of DNA samples that were > than Tm values of *P. falciparum*, positive both by 18S real-time PCR with only one melt in a curve and conventional PCR using *genus*-specific primers and, negative by conventional PCR using *P. falciparum*-specific primers. According to these definitions, we found that the melt curve means Tms (standard deviation) in clinical isolates were 60.5°C (0.6°C) for *P. falciparum *DNA amplicons and 64.6°C (1.8°C) for non-*P. falciparum *sp DNA amplicons. These results were closely related to melting temperature of 60.0 and 65.0°C for *P. falciparum *and non-*P. falciparum *sp respectively, that were estimated during primer and probe design using Oligo 4.0 software (LightCycler Probe Design Software, Roche). Tms of the *P. falciparum *or non-*P. falciparum *species were similar between geographic origin of parasite (Figure 4).

### Real-time PCR and conventional PCR assays

The results shown in Table 2 represent the comparison between 18S real-time PCR and conventional PCR assays. Ninety one out of 119 DNA samples (76.5%) were positive by both the 18S screening real-time PCR and conventional PCR using genus-specific primers, whereas 26 samples were negative by both methods, giving a concordance rate of 98.3% (CI_95%_: 89.3 – 107.6) between real-time PCR assay and conventional PCR using genus-specific primers (Kappa coefficient: 0.95, CI_95%_: 0.9 – 1). Two (1.7%) DNA samples positive by real-time PCR assay were negative by conventional PCR assay using genus-specific test (Table 2). Seventy one out of 119 DNA samples (59.7%) were positive by both the 18S real-time PCR and conventional PCR using *P. falciparum*-specific primers, whereas 26 samples were negative by both methods, giving a concordance rate of 96.6% (CI_95%_: 87.6 – 105.6) between real-time PCR assay and conventional PCR using genus-specific primers (Kappa coefficient: 0.91, CI_95%_: 0.8 – 1). Four (3.4%) DNA samples identified as *P. falciparum *by real-time PCR assay were negative by conventional PCR assay using *P. falciparum*-specific primers (Table 2).

**Table 2:** Detection of *Plasmodium *DNA by real-time and conventional PCR assays in patients at day of inclusion

		**Conventional PCR**, no. of patients			
		
		Genus-specific primers		*P. falciparum-*specific primers	
		
		POS^(a)^	NEG^(b)^	POS^(a)^	NEG^(b)^
**Real-Time PCR**	*P. falciparum*	71	1	68	4
	Non-*P. falciparum *sp.	17	1	0	18
	Mixed *Plasmodium *sp.^(c)^	3	0	3	0
	NEG^(b)^	0	26	0	26

^(a)^POS, posiive; ^(b)^NEG, negative; ^(c)^Mixed *Plasmodium *sp, *P. falciparum *+ non-*P. falciparum *sp.

### Real-time PCR and microscopy

Eighty six out of 119 DNA samples (72.3%) were positive by both the real-time PCR and microscopy, whereas 26 samples were negative by both methods, giving a concordance rate of 94.1% (CI_95%_: 85.1 – 103.1) between microscopy and real-time PCR for detection of any *Plasmodium *species (Table 3). A full agreement for *Plasmodium *sp. detection and species differentiation between both assays was found (Kappa coefficient: 0.70, CI_95%_: 0.6 – 0.8). Seven patients identified with Giemsa-stained blood smear as negative were positive with real-time PCR (five as *P. falciparum *infection and two as non-*P. falciparum *species). Eight DNA samples with undetermined species by microscopy were identified as *P. falciparum *by the real-time PCR (Table 3). These results were confirmed by conventional PCR assays using both genus- and *P. falciparum*-specific primers. The real-time PCR was able to detect mixed infection (*P. falciparum *+ non-*P. falciparum *species). Two patient samples positive for *P. ovale *(Giemsa-stained films) were recorded as samples harbouring *P. falciparum *and non-*P. falciparum *species with real-time PCR assay. One patient which microscopic result was mixed infection (*P. falciparum *and *P. vivax*, 2.6% and < 0.01% of parasitaemia, respectively) was also identified as mixed infection (*P. falciparum *+ non-*P. falciparum *sp.) with real-time PCR assay (Table 3). However, three patients with Giemsa-stained films positive for mixed infection (*P. falciparum *and *P. malariae*) were mono-infection with real-time PCR assay (2 patients with *P. falciparum *and one with non-*P. falciparum *sp.) (Table 3).

**Table 3:** Detection of *Plasmodium* species by microscopy and real-time PCR in patients at day of inclusion

		**Real-time PCR**, no. of patients			
		
		POS^(a)^			NEG^(b)^
			
		*P. falciparum*	non-*P. falciparum *sp	Mixed *Plasmodium *sp.^(c)^	
	
**Microscopy**	*P. falciparum*	54	0	0	0
	*P. ovale*	2	7	2	0
	*P. malariae*	0	2	0	0
	*P. vivax*	1	6	0	0
	*Plasmodium *sp	8	0	0	0
	Mixed infection	2	1	1	0
	NEG^(b)^	5	2	0	26

^(a)^POS, positive; ^(b)^NEG, negative; ^(c)^Mixed *Plasmodium *sp, *P. falciparum *+ non-*P. falciparum *sp.

## Discussion

A real-time PCR method using the FRET system for detection of malaria parasites, with simultaneous differentiation of the most threatening parasite *P. falciparum *from the three other *Plasmodium *species (*P. vivax, P. ovale and P. malariae*) was described. In order to increase the sensitivity of the assay, a species-specific region of the *Plasmodium *18S (small subunit) rRNA gene was targeted, as this gene contains multiple copies dispersed throughout the *Plasmodium *genome [36]. The real-time PCR results were compared with those of conventional PCR and microscopy.

The proposed protocol was performed in 90 minutes, including 30 minutes for DNA extraction, 15 minutes for mix preparation, and 45 minutes for amplification and results interpretation. In clinical isolates obtained from returned travellers, an equivalent concordance rate between real-time PCR assay and conventional PCR (≥ 97%) or microscopic (94%) was found. The specificity to detect *Plasmodium *sp. was 100% and the real-time PCR assay was able to detect parasitaemia less than 0.01% (microscopy examination). The proposed PCR method demonstrates a higher sensitivity to identify cryptic malaria infections. Seven febrile patients with negative Giemsa-stained films were positive with real-time PCR method. The analytical real-time PCR assay threshold was 0.5 compared to five parasites per PCR reaction with conventional PCR assay using genus-specific primers (the same primer used for real-time PCR assay). This sensitivity is more important than that was previously obtained by Swan and colleagues using similar approach [31], and correspond favourably to other published methods, specifically real-time PCR assays using TaqMan probes [23,25,27,37]. This high sensitivity is very important in term of biological diagnostic of imported malaria, as many returning travellers and migrants, sometimes under prophylactic medication or auto-medication, display febrile symptoms with very low-grade parasitaemia. In some case, parasite morphology is damaged due to exposition to prophylactic medication or auto-medication, making malaria biological diagnosis using microscopy more difficult.

The set of DNA primer and probe proposed in this study demonstrated a high specificity to differentiate *P. falciparum *from the three other *Plasmodium *species (*P. vivax, P. malariae *and *P. ovale*), based on the melting curve. Monoinfections were systematically identified, and real-time PCR has been even more effective than microscopy examination, identifying undetermined parasites, identified as *Plasmodium sp*. by microscopist due to the lack of specific form of parasites or morphological damages often resulting from previous or current antimalarial treatment. The real-time PCR assay was able to identify mixed infection (*P. falciparum *and non-*P. falciparum *sp.) and individual harbouring only gametocytes or gametocytes and asexual stages from different malaria *Plasmodium *species. Two patients who Giemsa-stained films were positive for *P. ovale *infection display mixed infection with real-time PCR assay. The validity of these results was supported by the clear visualization of two separate fluorescence peaks at different melting temperatures (59.7°C and 66.9°C, 59.5°C and 64.9°C, for the two assays), which correspond to the melting curve Tms of *P. falciparum *(60.5 ± 0.6°C) and non-*P. falciparum *sp. (64.6 ± 1.8°C). The detection of *P. falciparum *plus *P. malariae *mixed infection was not resolved. Three patients with mixed infection by microscopy (*P. falciparum *and *P. malariae *with parasitaemia density of *P. malariae *< 0.01%) were identified as *P. falciparum *mono-infection (n = 2) or non-*P. falciparum *sp. mono-infection (n = 1) with real-time PCR assay. This discrepancy was also observed in experimental mixed infection with *P. falciparum *and *P. malariae*. The low number of included patients harbouring mixed infection make that more experiments need to be conducted in order to precise detection limit of parasites in the case of mixed infections. Precise identification of the presence of *P. falciparum *associated or not with other *Plasmodium *species is a critical issue for patient care and treatment outcome, since i) *P. falciparum *infections are potentially fatal and ii) drug resistance occur at a high level compared to other species.

Geographically diverse malaria isolates obtained from returning febrile travellers and migrants were analysed to ensure that the assay would reliably differentiate *P. falciparum *from the three other species despite the parasite origin. The result indicates that the single base pair mismatched which allowed differentiation of *P. falciparum *to the three other species is well conserved between geographic regions of parasite which was evaluated. Single base differences theoretically may also exist between strains within a species from different geographic regions, but our results indicate that no variance was found among individual isolates. Additional specimens from other regions around the world would be required to confirm these observations.

Recently, *Plasmodium knowlesi*, a species that has morphological similarities with *P. malariae*, has been identified as a human pathogen in patients from Malaysia [38]. Sequence analysis indicates that this species may also be amplified by using the set of primer and probe proposed in this study, and the expected Tm will be similar to that of non-*P. falciparum *species. Further confirmations are needed by testing *P. knowlesi *specimens with this real-time PCR method.

## Conclusion

A rapid, FRET real-time PCR assay using one set of primer and probe for the diagnosis of *Plasmodium *sp. in febrile returning travellers and migrants was described. An important advantage of this approach is the higher sensitivity and specificity for the detection of *Plasmodium *sp. and to differentiate *P. falciparum *from the three other malaria parasite species (*P. vivax, P. malariae *and *P. ovale*), based on the melting. Concurrently to microscopic examination, such results can be used to ensure rapid treatment administration and proper follow-up of the malaria attacks by the practitioner. Except for *P. falciparum *and *P. malariae *mixed infection, the ability to provide accurate species identification in the same sample used for detection of the parasite is also a greatest attribute of this method. The proposed protocol is performed through automated extraction, amplification and interpretation allowing access to any laboratory technician without specific knowledge in the field of malaria. Although the relatively high cost of real-time PCR technology may preclude its use in resource-poor clinics, its performance characteristics, combined with its rapid results, suggest that it may be a useful diagnostic adjunct in industrialized countries.

## Authors' contributions

IS participated in conceptualising the real-time PCR assay, contributed to the design of the study, conducted the PCR methods and wrote the first draft of the manuscript. LM conducted the PCR methods and participated to drafting of the manuscript. SB, PM and HF participated in conceptualising the real-time PCR method, contributed to the design of the study and critically reviewed the manuscript. FF, PF and PV processed the isolates and read slides and critically reviewed the manuscript. CR and TP contributed to the enrolment of patients and critically reviewed the manuscript. DM contributed to the design of the study, supervised the enrolment of patients and critically reviewed the manuscript. All authors read and approved the final manuscript.
